# Characterization of the Self-Resistance Mechanism to Dityromycin in the *Streptomyces* Producer Strain

**DOI:** 10.1128/mSphere.00554-19

**Published:** 2019-09-25

**Authors:** Attilio Fabbretti, Retina Çapuni, Anna Maria Giuliodori, Lucia Cimarelli, Antonino Miano, Valerio Napolioni, Anna La Teana, Roberto Spurio

**Affiliations:** a Laboratory of Genetics, School of Biosciences and Veterinary Medicine, University of Camerino, Camerino, Italy; b Structural Biology Unit, CIC bioGUNE, Derio, Bizkaia, Spain; c Department of Neurology and Neurological Sciences, Stanford University School of Medicine, Stanford, California, USA; d Department of Life and Environmental Sciences, Polytechnic University of Marche, Ancona, Italy; University of Nebraska Medical Center

**Keywords:** self-resistance, antibiotic, ribosomal protein S12, translation

## Abstract

Dityromycin is a peptide antibiotic isolated from the culture broth of the soil microorganism *Streptomyces* sp. strain AM-2504. Recent structural studies have shown that dityromycin targets the ribosomal protein S12 in the 30S ribosomal subunit, inhibiting translocation. Herein, by using *in vitro* protein synthesis assays, we identified the resistance mechanism of the producer strain to the secondary metabolite dityromycin. The results show that the self-resistance mechanism of the *Streptomyces* sp. strain AM-2504 is due to a specific modification of the ribosome. In particular, two amino acid substitutions, located in a highly conserved region of the S12 protein corresponding to the binding site of the antibiotic, were found. These mutations cause a substantial loss of affinity of the dityromycin for the 30S ribosomal subunit, protecting the producer strain from the toxic effect of the antibiotic. In addition to providing a detailed description of the first mechanism of self-resistance based on a mutated ribosomal protein, this work demonstrates that the molecular determinants of the dityromycin resistance identified in *Streptomyces* can be transferred to Escherichia coli ribosomes, where they can trigger the same antibiotic resistance mechanism found in the producer strain.

**IMPORTANCE** The World Health Organization has identified antimicrobial resistance as a substantial threat to human health. Because of the emergence of pathogenic bacteria resistant to multiple antibiotics worldwide, there is a need to identify the mode of action of antibiotics and to unravel the basic mechanisms responsible for drug resistance. Antibiotic producers’ microorganisms can protect themselves from the toxic effect of the drug using different strategies; one of the most common involves the modification of the antibiotic’s target site. In this work, we report a detailed analysis of the molecular mechanism, based on protein modification, devised by the soil microorganism *Streptomyces* sp. strain AM-2504 to protect itself from the activity of the peptide antibiotic dityromycin. Furthermore, we demonstrate that this mechanism can be reproduced in E. coli, thereby eliciting antibiotic resistance in this human commensal bacterium.

## Abstract (precis)

The World Health Organization has identified antimicrobial resistance as a substantial threat to human health. Because of the emergence of pathogenic bacteria resistant to multiple antibiotics worldwide, there is a need to identify the mode of action of antibiotics and to unravel the basic mechanisms responsible for drug resistance. Antibiotic producers’ microorganisms can protect themselves from the toxic effect of the drug using different strategies; one of the most common involves the modification of the antibiotic’s target site. In this work, we report a detailed analysis of the molecular mechanism, based on protein modification, devised by the soil microorganism *Streptomyces* sp. strain AM-2504 to protect itself from the activity of the peptide antibiotic dityromycin. Furthermore, we demonstrate that this mechanism can be reproduced in E. coli, thereby eliciting antibiotic resistance in this human commensal bacterium.

## INTRODUCTION

The emergence of multidrug-resistant (MDR) bacteria is a growing problem that represents one of the top three threats to global public health worldwide (1–4). Moreover, it has been estimated that approximately 70% of infection-causing bacteria are already resistant to at least one antibiotic available in routine clinical practice. Therefore, all the efforts to provide a detailed molecular picture of a specific resistance mechanism can contribute positively to improve our understanding of antimicrobial resistance (AMR) and its dynamics.

**FIG 1:**
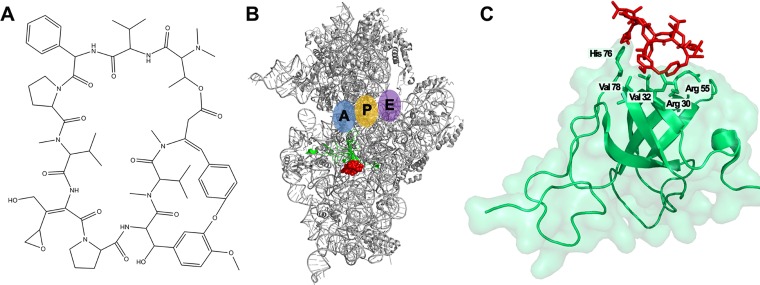
Interaction of dityromycin with the 30S ribosomal subunit. (A) Chemical structure of the antibiotic dityromycin. (B) Three-dimensional (3D) structure of the 30S ribosomal subunit (gray) and localization of dityromycin (red) in contact with the ribosomal protein S12 (green). A, P, and E tRNA binding sites are indicated in blue, yellow, and violet, respectively. (C) Close-up of dityromycin (red) bound to S12 (green) missing the C-terminal domain. The amino acid residues of S12 involved in the interaction with the antibiotic are also indicated. (Modified from reference 9.)

Dityromycin is a cyclic decapeptide antibiotic (Fig. 1A) discovered as a secondary metabolite produced by *Streptomyces* sp. strain AM-2504 (5, 6). Its structure is almost identical to that of GE82832, a translocation inhibitor produced by *Streptosporangium* spp. (7–9). Both molecules interact with the same ribosomal site and display the same mechanism of action, even if they are produced by different microorganisms. The interaction of dityromycin with the ribosome is peculiar compared to other ribosomal inhibitors, since it interacts exclusively with the ribosomal protein S12 without a direct interaction with the 16S rRNA (Fig. 1B and C) (8, 9). Indeed, the binding of dityromycin to the ribosome is mediated by its interaction with five amino acids at the ribosomal protein S12, namely, arginine 30, valine 32, arginine 55, histidine 76, and valine 78 (Fig. 1C). Dityromycin inhibits protein synthesis by blocking the translocation of tRNA through the ribosome, while it has almost no effect on the accommodation of aminoacyl-tRNA into the P-site or the A-site, in the context of either the 30S ribosomal subunit or the 70S ribosome (7). Recent studies have shown that dityromycin blocks the EF-G-dependent translocation of peptidyl tRNA and mRNA without preventing the ribosomal binding of the elongation factor. In particular, dityromycin hampers the interaction between domain III of EF-G and protein S12, which is located near the decoding center of the ribosome, at the interface of two subunits (9, 10).

A strict requirement to ensure that the antibiotic producer cell protects itself when the active metabolites are produced, accumulated, and released is to possess a self-resistance mechanism(s) (11). Microorganisms can adopt several strategies to develop self-resistance, such as (i) chemical modification of the target, as in the case of rRNA methylation or mutation (12, 13); (ii) activation of efflux pumps (14, 15); (iii) sequestration of the natural product by proteins keeping the active compound in a bound and inactive state (16, 17); (iv) production of the antibiotic in a prodrug form, activated through selective cleavage by peptidases found in the periplasmic space, and subsequently released outside the cell (18, 19); and (v) oxidative inactivation of the matured prodrug following export (20).

Biochemical and genetic similarities demonstrate that such resistance mechanisms have prefigured those found subsequently in antibiotic-resistant pathogens, which have acquired specific genes or gene clusters originally developed by antibiotic producers (21).

In this work, we have investigated the self-resistance mechanism developed by *Streptomyces* sp. strain AM-2504 toward the antibiotic activity of its natural product dityromycin. To prevent self-toxicity, this microorganism has evolved a strategy based on the production of a modified and self-resistant ribosomal protein variant. Our approach consisted in analyzing three aspects, namely, identification and biochemical characterization of the self-resistance mechanism, characterization of the amino acid residues of the ribosomal protein S12 involved in resistance by the heterologous expression of site-directed mutants in Escherichia coli, and a phylogenetic analysis of the dityromycin binding pocket in the ribosomal protein S12.

## RESULTS

### Identification of the mechanism conferring self-resistance.

To determine the self-resistance mechanism of *Streptomyces* sp. strain AM-2504 to dityromycin, we set up a simple experiment evaluating whether the resistance mechanism relied on possible modifications/secretion of the drug (prodrug or efflux pumps) or was based on mutations/methylations/modifications of the target.

**FIG 2:**
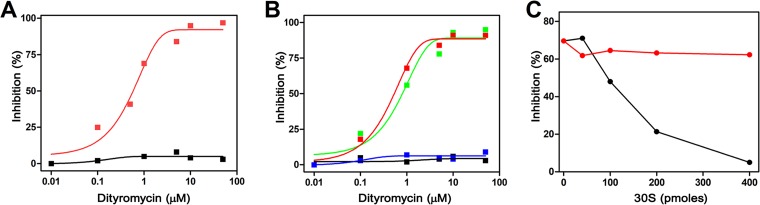
Identification of the self-resistance mechanism. (A and B) Effects of increasing concentration of dityromycin on poly(U) *in vitro* translation based on S30 cell extract of E. coli (red) or *Streptomyces* sp. strain AM-2504 (black) (A) or crisscrossed assays using post-ribosomal supernatant (S100) and 70S ribosomes of *Streptomyces* sp. strain AM-2504 or of E. coli (B). *Streptomyces* 70S incubated with E. coli S100 or *Streptomyces* S100 is indicated in blue or black, respectively. E. coli 70S incubated with either E. coli S100 or *Streptomyces* S100 is indicated in red or green, respectively. (C) Residual translation inhibition activity of the supernatants obtained from ultracentrifugation of 30S ribosomes preincubated with dityromycin. After centrifugation of increasing amounts of E. coli 30S (black) and *Streptomyces* sp. strain AM-2504 30S (red), the residual inhibition activity of the supernatants was determined in a poly(U) translation test (further details are provided in Materials and Methods).

A translational test programmed with poly(U) was used to compare cell extracts prepared from either E. coli (S30_E.coli_) or *Streptomyces* sp. strain AM-2504 cells (S30_Str_) in the presence or absence of the antibiotic. Dityromycin, an antibiotic targeting the translation elongation phase, completely inhibited protein synthesis with S30_E.coli_ at concentrations ranging between 1 and 10 μM, whereas it did not show any effect on the poly(U) translation carried out with S30_Str_ (Fig. 2A). The lack of inhibition with the S30_Str_ extract clearly excluded prodrug or efflux pumps as a possible self-resistance mechanism.

To test whether this self-resistance mechanism was caused by protective mutations in the ribosomes or by an enzymatic activity able to modify/deactivate the antibiotic, 70S ribosomes were separated by ultracentrifugation from the post-ribosomal supernatant (S100) and the fractions were tested by crossed *in vitro* poly(U) translation assays. In particular, the effect of dityromycin on *Streptomyces* 70*S* ribosomes (70S_Str_) was assayed in the presence of E. coli S100 (S100_E.coli_). *Vice versa*, *Streptomyces* S100 extract (S100_Str_) was tested with E. coli 70S ribosomes (70S_E.coli_). The results of this experiment (Fig. 2B) show that the reaction mixtures consisting of S100_Str_ plus 70S_E.coli_ and S100_E.coli_ plus 70S_E.coli_ were totally inhibited by dityromycin, whereas those formed by S100_E.coli_ plus 70S_Str_ and S100_Str_ plus 70S_Str_ were not. These results strongly indicate that the modifications causing the self-resistance of the producer strain lie on the ribosome.

Since the target of dityromycin is the ribosomal protein S12 (9), 30S ribosomal subunits from both E. coli and *Streptomyces* sp. strain AM-2504 were purified. Increasing concentrations of these isolated particles were used in a pulldown assay conducted in the presence of a fixed concentration of the antibiotic. After ultracentrifugation at 300,000 × *g*, the supernatants were tested in a translational system based on E. coli cell extracts programmed with poly(U) to detect the residual presence of unbound dityromycin (see Materials and Methods). As reported in Fig. 2C, the supernatant resulting from the pulldown of E. coli 30S subunits incubated with dityromycin did not interfere with poly(Phe) synthesis. Conversely, the supernatant obtained after centrifugation of *Streptomyces* 30S subunits incubated with dityromycin inhibited the reaction. This result indicates that dityromycin is not bound by the *Streptomyces* 30S subunits and is not pulled down during ultracentrifugation, even at high concentrations of ribosomes. In contrast, dityromycin stably interacts with the E. coli 30S subunits and is thus removed from the supernatant during the centrifugation step (Fig. 2C). In conclusion, the present results point to a self-resistance mechanism based on a mutation/modification residing in the small ribosomal subunit.

### Sequencing of the S12 gene reveals three amino acid substitutions in the dityromycin binding site.

As shown in the crystallographic structure of Fig. 1, dityromycin binds the 30S subunit by interacting exclusively with five critical positions of the ribosomal protein S12, namely, Arg30, Val32, Arg55, His76, and Val78 (Fig. 1C). To clarify the role played by S12 in reducing the binding affinity of dityromycin to the 30S_Str_ subunit (Fig. 2), we sequenced the *rpsL* gene, encoding the S12 protein of *Streptomyces* sp. strain AM-2504. In addition, we sequenced the entire genome of the producer strain, thus obtaining its first annotated draft genome sequence (22). The comparison of *Streptomyces* sp. strain AM-2504 genome with the sequences available in the NCBI genome data bank allowed us to verify that the strain named AM-2504 described in this study exhibits 98.6% nucleotide identity with Streptomyces kasugaensis. Therefore, it is conceivable that *Streptomyces* sp. strain AM-2504 can be classified as S. kasugaensis.

**FIG 3:**
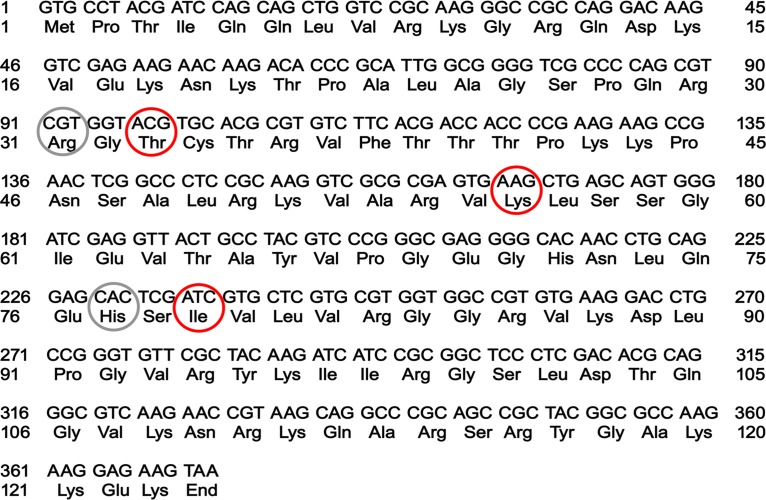
Sequence of the *rpsL* gene of *Streptomyces* sp. strain AM-2504. Nucleotide and amino acid sequences of ribosomal protein S12 *of Streptomyces* sp. strain AM-2504. The amino acids involved in the interaction with the antibiotic in dityromycin-sensitive ribosomes are circled. Amino acids involved in the self-resistance mechanism are circled in red.

DNA sequence data demonstrate that three out of five amino acids located in the dityromycin binding site of S12, namely, Val32Thr, Arg55Lys, and Val78Ile, are different from those found in the model organism E. coli (Fig. 3). These substitutions are likely the molecular determinants of the self-resistance mechanism to the antibiotic, preventing dityromycin binding to the ribosome (Fig. 2C).

### Expression of the S12 mutant proteins in E. coli confers resistance to dityromycin.

**FIG 4:**
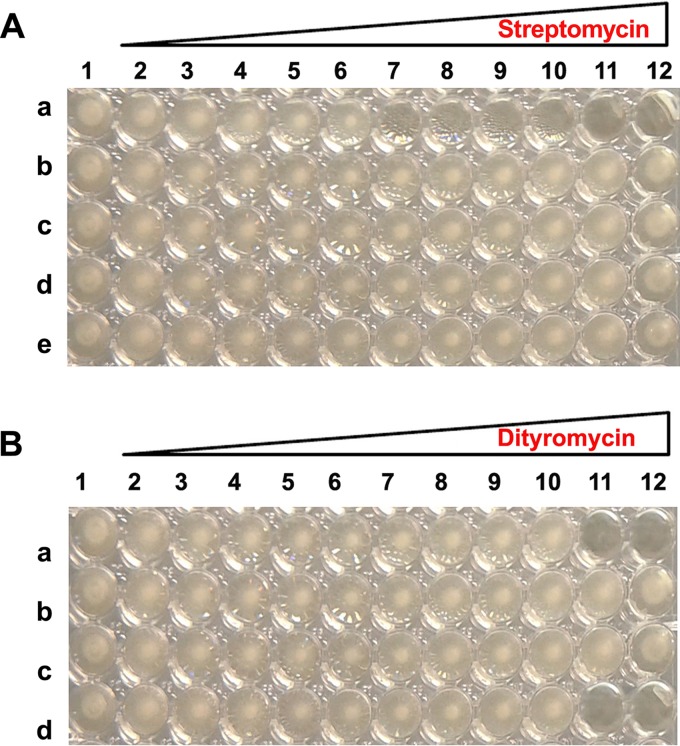
Mutations in ribosomal protein S12 confer resistance to dityromycin in E. coli. (A) Determination of MIC of streptomycin on E. coli BL21(DE3)/pLysS (a) transformed with pET11a-rpsl_K42R_ (b) or with pET11a-rpsl_K42R_ carrying the site-directed mutation of the dityromycin binding pocket V32T (c), R55K (d), or V78I (e). (B) Determination of MIC of dityromycin on E. coli BL21(DE3)*/*pLysS transformed with pET11a-rpsl_K42R_ (a) or with pET11a-rpsl_K42R_ carrying the site-directed mutation of the dityromycin binding pocket V32T (b), R55K (c), or V78I (d). The streptomycin concentrations were 0, 0.5, 1.0, 2.0, 4.0, 8.0, 16.0, 32.0, 64.0, 128.0, 256.0, and 512.0 μg/ml in lanes 1 to 12, respectively. The dityromycin concentrations were 0, 0.39, 0.78, 1.56, 3.12, 6.25, 12.5, 25.0, 50.0, 100.0, 200.0, and 400.0 μg/ml from lanes 1 to 12, respectively.

To verify whether these mutations are responsible for the self-resistance of the producer strain, single substitutions at Val32, Arg55, and Val78 were introduced into the E. coli *rpsL* gene, and the mutant genes were provided in *trans* to the E. coli BL21 strain grown in the presence of increasing concentrations of dityromycin. The experimental system was based on gene expression from the high-copy-number plasmid pET11a, which contained the *rpsL* gene isolated from E. coli MC4100 carrying the original mutation Lys42Arg, which confers resistance to the antibiotic streptomycin. As shown in Fig. 4A, E. coli cells expressing a wild-type copy of S12, in the absence of the plasmid, are sensitive to streptomycin, but they become resistant once transformed. Moreover, the presence of either Val32Thr, Arg55Lys or Val78Ile substitutions did not affect the streptomycin-resistant phenotype. This demonstrates that the ribosomes effectively incorporate the S12 protein encoded by the plasmid and respond to the mutant genetic background supplied in *trans*. Furthermore, the results shown in Fig. 4B demonstrate that the ribosomal protein S12 carrying the Val32Thr or Arg55Lys substitution confer dityromycin resistance to E. coli, whereas the cells expressing the point mutation Val78Ile proved to be susceptible to this antibiotic.

## DISCUSSION

The growing number of bacterial pathogens uniquely adapted to survive in the presence of one or more antibiotics is an extremely serious threat to global health. The term “antibiotic resistome,” a large repertoire of genes that has been originally developed by bacteria and is currently remodeled by the selective pressure imposed by drug use/abuse, has been coined to describe the genetic determinants involved in antibiotic resistance (23, 24).

Resistance to antibiotics is tightly linked to their production, since microorganisms producing these molecules must protect themselves against their action. In many cases, mechanisms of self-resistance have been shown to be mechanistically similar to the ones identified in clinical pathogens (25). Resistance genes are often carried by the same gene cluster of the antibiotic biosynthetic enzymes, and their expression is temporally coupled. Indeed, antibiotic producer cells need to express resistance determinants prior to, or concomitantly with, synthesis of the antibiotic molecule (26, 27). In recent years, the analysis of several genomes suggested that microorganisms produce only a fraction of the secondary metabolites that they encode, and new approaches have been used to identify biosynthetic gene clusters whose expression can be induced. The colocalization of resistance genes and biosynthetic genes in the genomes of underexploited groups of secondary metabolite producers, for example, has been used in pursuit of new biosynthetic pathways. This genome mining approach, guided by the search for self-resistant determinants, has been successful for topoisomerase inhibitors whose biosynthetic gene cluster has been identified by looking for putative pentapeptide repeat proteins, which are known to confer self-resistance to these molecules (28).

More than half of the antibiotics currently used inhibit protein synthesis by binding to the functional centers of the ribosome. Recently, important insights into the mechanism of these antibiotics have been obtained by X-ray crystallography and cryo-electron microscopy (Cryo-EM) analysis, opening the possibility of developing new molecules by structure-based drug design (29–36).

In this work, we characterized the mechanism of self-resistance of the dityromycin producer *Streptomyces* sp. strain AM-2504. Using E. coli and *Streptomyces*-based *in vitro* translation assays in combination with an ultracentrifugation pulldown assay, we demonstrated that the resistance determinants are localized in the *Streptomyces* 30S ribosomal subunit. Since the target of dityromycin is the ribosomal protein S12, we sequenced the gene coding for *Streptomyces* S12 and compared it with its E. coli homolog. Three amino acids in the *Streptomyces* protein (out of the five involved in the interaction with the antibiotic) differed from the E. coli protein sequence. Therefore, we suggest that the molecular basis underlying the self-resistance mechanism could be the reduced affinity of the antibiotic toward its target due to the amino acid substitutions on the ribosomal protein S12. To confirm this hypothesis, we introduced the observed amino acid substitutions into a plasmid-borne copy of E. coli *rpsL* gene harboring another mutation conferring resistance to the antibiotic streptomycin. The observed streptomycin resistance phenotype provides explicit, albeit indirect, evidence that the plasmid gene encoding ribosomal protein S12 is effectively incorporated into the 30S subunits. Even though the setup of the assay did not allow strict quantification of the number of copies of mutant protein incorporated, it showed unequivocally that this incorporation took place and that plasmid-encoded S12 bearing two specific mutations in the dityromycin binding site conferred resistance to the antibiotic. The results shown in Fig. 4B clearly indicate the effects of amino acid replacements at the three key sites of ribosomal protein S12 involved in the binding of the antibiotic. In particular, at position 32, the substitution of the nonpolar aliphatic valine with the polar amino acid threonine interferes with the hydrophobic interaction established with the dityrosine group of dityromycin according to the crystal structure of the dityromycin-30S complex. It has been pointed out by crystallographic analysis that the arginine at position 55 packs against the *N*,*N*-dimethyl valine of the antibiotic (9). Our results strongly indicate that the replacement of a positively charged side chain, as in the case of lysine, is not sufficient to maintain the correct structure of the binding site, which presumably requires the chemical properties of the guanidinium group of arginine. This finding is consistent with the high specificity demonstrated by the arginine side chain in the interaction of proteins with their substrates (37, 38). On the other hand, we demonstrate that the substitution of Val78 with isoleucine does not interfere with the interaction of dityromycin with S12.

To identify the degree of conservation of the amino acid residues responsible for dityromycin resistance, we aligned the S12 sequences to 72 *Streptomyces* spp. randomly selected from the NCBI database. The alignment shows that, of the three residues considered, only Ile78 (Val in E. coli) is conserved in all *Streptomyces* sequences analyzed. The other two key residues are quite rare, with Thr32 (Val in E. coli) reported only in 7 out of 72 genomes (*Streptomyces antioxidans*, *S. autoliticus*, *S. gilvosporeus*, *S. iranensis*, *S. malaysiensis*, *S. natalensis*, and *S. violaceusniger*) and Lys55 (Arg in E. coli) in only two strains, *S. gilvosporeus* and *S. natalensis*. Therefore, the results of this comparative analysis show that only 2 of 72 *Streptomyces* strains share the same S12 residues conferring resistance to dityromycin as *Streptomyces* sp. strain AM-2504 (see Fig. S1 in the supplemental material). When the comparative analysis was extended to 30 bacterial strains randomly covering all relevant branches of the phylogenetic tree, it was found that all selected bacteria display a valine at position 32, embedded in a stretch of four amino acids (RGVC), which is highly conserved and it is part of the “core” of a pseudo beta-barrel of S12 (Fig. S2A and S2B). The other two residues are less conserved, since Lys55 and Ile78 are found in 4 strains and 11 bacterial strains, respectively.

10.1128/mSphere.00554-19.1 FIG S1 Alignment of the amino acid sequence of ribosomal protein S12 from 72 *Streptomyces* strains. The arrows indicate the amino acids that are involved in dityromycin binding that are different from those in the E. coli counterpart. Download FIG S1, JPG file, 2.2 MB. Copyright © 2019 Fabbretti et al. 2019 Fabbretti et al. This content is distributed under the terms of the Creative Commons Attribution 4.0 International license.

10.1128/mSphere.00554-19.2 FIG S2 (A) Alignment of the amino acid sequence of ribosomal protein S12 from 30 bacterial species. The arrows indicate the amino acids involved in dityromycin binding, and the red box indicates the highly conserved RGVC sequence motif. (B) Phylogenetic tree representing the relatedness of ribosomal protein S12 of the bacterial species reported in panel A. Download FIG S2, TIF file, 2.6 MB. Copyright © 2019 Fabbretti et al. 2019 Fabbretti et al. This content is distributed under the terms of the Creative Commons Attribution 4.0 International license.

To our knowledge, the results of this work represent the first example of a self-resistance mechanism mediated by a ribosomal protein in the producer strain. Resistance mechanisms identified by isolation of single mutations are extremely rare among ribosomal proteins. Point mutations in *rpsL*, the gene encoding S12, have been identified as the key factor conferring streptomycin resistance (39) and at the same time activate the expression of cryptic antibiotics, like the blue-colored actinorodhin (40, 41). This intriguing finding, which is the first reported evidence of an effect induced on gene expression by a point mutation located on a ribosomal protein, is still lacking a detailed explanation at the molecular level. A few other examples include protein L3, where a single mutation has been shown to confer resistance to tiamulin, the semisynthetic derivative of pleuromutiline, reducing its binding affinity for the ribosome and mutations in L16 conferring resistance to the oligosaccharide antimicrobial agents avilamycin (42) and evernimicin (43). In Pasteurella multocida, a bacterium responsible for zoonotic diseases, a combination of amino acid changes on ribosomal protein S5 (point mutation at Ser32 and amino acid deletion at Phe33) and a single transversion in helix 34 of 16S rRNA have been identified as the molecular determinants of spectinomycin resistance (44).

In summary, we can conclude that *Streptomyces* sp. strain AM-2504 has developed a successful strategy to protect its own ribosomes from the effect of dityromycin, combining the effects of three amino acid substitutions, which occur as isolated point mutations in other Actinomyces strains and are compatible with the structural function of ribosomal protein S12. Although this resistance mechanism does not fall into the category of antibiotic resistance transfer and spreading, in a strict sense, the characterization of this strategy at the molecular level adds a new piece to the complex puzzle underlying the coevolution of antibiotic production and self-resistance mechanisms.

## MATERIALS AND METHODS

### Media.

ISP2 medium (sporulation agar) consists of 0.4% yeast extract, 0.4% glucose, 1% malt extract, and 2% agar. T1 medium contains 1% glucose, 2% starch, 0.5% yeast extract, 0.5% peptone, and 0.4% CaCO_3_. JM medium consists of 0.1% yeast extract, 0.1% MgCl_2_ (6H_2_O), 0.3% Tryptone Soya, and 10% sucrose. In all cases, the pH of the medium was adjusted to 7.0 prior to autoclaving. Luria-Bertani medium was used for the growth of Escherichia coli strains utilized for propagation of plasmids and for the preparation of ribosomes and cell extracts.

### Culture conditions of *Streptomyces* sp. strain AM-2504.

For the production and isolation of spores, *Streptomyces* sp. strain AM-2504 was grown in ISP2 medium for 7 to 10 days at 28°C. The white aerial mycelium, consisting of spores, was stripped from the upper layer of the plate using a spatula and dispersed into 1 ml of sterile water. The spore suspension was filtered through a wad of absorbent cotton, and the spores were concentrated by centrifugation at 4,000 rpm for 15 min. The spores were resuspended in 100 μl of 20% glycerol and stored at –20°C.

### Genomic DNA extraction.

*Streptomyces* spores (20 μl) were inoculated in 20 ml of JM medium at 28°C for 18 h with aeration. Cells were collected by centrifugation, and genomic DNA was extracted using the Invisorb spin plant minikit (Invitek).

### DNA amplification and sequence of *rpsL* from *Streptomyces* sp.

Oligonucleotide primers GSF1 and GSR1 (Table 1), designed to cover the coding region of the *rpsL* gene from *Streptomyces*, were used to amplify by PCR the target gene using genomic DNA as the template. The DNA sequence of the amplicon, covering approximately 95% of the coding region, was analyzed to identify the amino acid variants carried by the antibiotic producer strain.

**TABLE 1:** Oligonucleotide primers used in this study

Primer	DNA sequence (5′→3′)^*a*^	Target
Forward mutagenic primer	CCGCAAAAACGTGGCACATGTACTCGTG	Amino acid substitution Val32→Thr32
Reverse mutagenic primer	CACGAGTACATGTGCCACGTTTTTGCGG	Amino acid substitution Val32→Thr32
Forward mutagenic primer	GTATGCCGTGTTAAACTGACTAACGGTTTCG	Amino acid substitution Arg55→Lys55
Reverse mutagenic primer	CGAAACCGTTAGTCAGTTTAACACGGCATAC	Amino acid substitution Arg55→Lys55
Forward mutagenic primer	CAGGAGCACTCCATAATCCTGATCCGTG	Amino acid substitution Val78→Ile78
Reverse mutagenic primer	CACGGATCAGGATTATGGAGTGCTCCTG	Amino acid substitution Val78→Ile78
*rpsL*-specific forward primer (GSF1)	GTGCCTACGATCCAGCAGCTG	*Streptomyces rpsL* gene
*rpsL*-specific reverse primer (GSR1)	TTACTTCTCCTTCTTGGCGCCG	*Streptomyces rpsL* gene

a The underlined nucleotides are the mutant triplet introduced by site-directed mutagenesis.

### Site-directed mutagenesis.

Mutations in the E. coli *rpsL* gene were obtained using the QuikChange XL kit (Stratagene) and, as the template for the PCR, the plasmid vector pET11a carrying the coding sequence of S12, derived from E. coli MC4100 (*rpsL150* [Str^r^]). The DNA sequences of the oligonucleotide primers used to introduce codon variants are indicated in Table 1. E. coli DH5 cells were transformed with the mutagenized plasmids, and Sanger DNA sequencing confirmed the presence of mutated nucleotides.

### Preparation of E. coli and *Streptomyces* S30 cell extracts.

E. coli MRE600 cells were grown at 37°C in LB medium until they reached mid-log phase, while *Streptomyces* cells were grown at 28°C in T1 medium for 45 h. Both cell types were disrupted by grinding with precooled alumina in a chilled mortar and processed following a previously described protocol (45). Both S30 extracts were extensively dialyzed against a buffer containing 10 mM Tris-HCl (pH 7.7), 10 mM Mg acetate, 60 mM NH_4_Cl, and 0.5 mM dithiothreitol (DTT) and stored in small aliquots at –80°C.

### Isolation of ribosomes and ribosomal subunits.

The crude extract S30 was subjected to ultracentrifugation at 100,000 × *g* for 17 h at 4°C. The supernatant, called S100, was stored at –80°C, while the pellet, consisting of 70S ribosomes, was resuspended and split in two aliquots, one for *in vitro* assays and one for preparation of 30S and 50S ribosomal subunits. 70S ribosomes were dissociated into subunits at 1 mM Mg^2+^ concentration by ultracentrifugation at 25,000 rpm for 17h at 4°C in a swing bucket rotor (SW28), on a 10% to 30% sucrose density gradient (45).

### *In vitro* translation assays.

Cell-free systems programmed with either poly(U) or 027 model mRNA were used to investigate the effect of dityromycin on the bacterial translational apparatus. Translation activity tests were performed using either S30 extracts or S100 extracts and 70S ribosomes, or crisscrossed combinations of E. coli and *Streptomyces* sp. S100 extracts and 70S ribosomes (46).

### Ribosome pulldown assay.

The binding of dityromycin to either *Streptomyces* or E. coli 30S ribosomal subunits was monitored by an ultracentrifugation-based assay. Increasing concentrations of 30S subunits were incubated for 10 min at room temperature with a fixed amount of antibiotic (1 μM) in 90 μl of a solution consisting of 10 mM Tris-HCl (pH 7.7), 10 mM Mg acetate, 60 mM NH_4_Cl, and 0.5 mM DTT. After ultracentrifugation in a S-100 AT-3 rotor at 100,000 rpm for 1 h at 4°C, aliquots of 20 μl of the supernatant were removed and tested in poly(U)-dependent *in vitro* translation assays.

### Determination of MIC.

To verify the incorporation of S12 mutants in E. coli ribosomes, a MIC plate (5 rows × 12 wells) was set up with increasing concentrations of streptomycin, ranging from 0.0 μg/μl to 512.0 μg/μl. Each well of the plate contained 190 μl of E. coli BL21(DE3)/pLysS cell culture. The plate rows contained the following: (i) no-plasmid control, (ii) pET11a expression vector with *rpsL150*, (iii) mutant V32/T32, (iv) mutant R55/K55, and (v) mutant V78/I78.

A MIC plate (4 rows × 12 wells) with the four tester strains and a gradient of dityromycin (from 0.0 μg/μl to 400 μg/μl) was used to estimate the ability of the mutant proteins to counteract the presence of the antibiotic. Further details are provided in the legend to Fig. 4.

## References

[1] World Health Organization. 2000 Overcoming antibiotic resistance. World Health Organization Report on Infectious Diseases 2000. World Health Organization, Geneva, Switzerland.

[2] Singer RS, Finch R, Wegener HC, Bywater R, Walters J, Lipsitch M 2003 Antibiotic resistance – the interplay between antibiotic use in animals and human beings. Lancet Infect Dis 3:47–51. doi:10.1016/S1473-3099(03)00490-0. 12505035

[3] Review on Antimicrobial Resistance. 2016 Tackling drug-resistant infections globally: final report and recommendations. The Review on Antimicrobial Resistance, London, United Kingdom.

[4] Friedrich AW 2019 Control of hospital acquired infections and antimicrobial resistance in Europe: the way to go. Wien Med Wochenschr 169:25–30. doi:10.1007/s10354-018-0676-5. PMC6373234 30623278

[5] Omura S, Iwai Y, Hirano A, Awaya J, Suzuki Y, Matsumoto K 1977 A new antibiotic, AM-2504. Agric Biol Chem 41:1827–1828. doi:10.1271/bbb1961.41.1827.

[6] Thesima T, Nishikawa M, Kubota I, Shiba T, Iwai W, Omura S 1988 The structure of an antibiotic, dityromycin. Tetrahedon Lett 29:1963–1966. doi:10.1016/S0040-4039(00)82090-0.

[7] Brandi L, Fabbretti A, Di Stefano M, Lazzarini A, Abbondi M, Gualerzi CO 2006 Characterization of GE82832, a peptide inhibitor of translocation interacting with bacterial 30S subunit. RNA 12:1262–1270. doi:10.1261/rna.61206. 16699167 PMC1484444

[8] Brandi L, Maffioli S, Donadio S, Quaglia F, Sette M, Milón P, Gualerzi CO, Fabbretti A 2012 Structural and functional characterization of the bacterial translocation inhibitor GE82832. FEBS Lett 586:3373–3378. doi:10.1016/j.febslet.2012.07.040. 22841550

[9] Bulkley D, Brandi L, Polikanov YS, Fabbretti A, O’Connor M, Gualerzi CO, Steitz TA 2014 The antibiotics dityromycin and GE82832 bind protein S12 and block EF-G-catalyzed translocation. Cell Rep 6:357–365. doi:10.1016/j.celrep.2013.12.024. 24412368 PMC5331365

[10] Demirci H, Wang L, Murphy FV, Murphy EL, Carr JF, Blanchard SC, Jogl G, Dahlberg AE, Gregory ST 2013 The central role of protein S12 in organizing the structure of the decoding site of the ribosome. RNA 19:1791–1801. doi:10.1261/rna.040030.113. 24152548 PMC3884664

[11] Hopwood DA 2007 How do antibiotic-producing bacteria ensure their self-resistance before antibiotic biosynthesis incapacitates them? Mol Microbiol 63:937–940. doi:10.1111/j.1365-2958.2006.05584.x. 17238916

[12] Liu M, Douthwaite S 2002 Methylation at nucleotide G745 or G748 in 23S rRNA distinguishes Gram-negative from Gram-positive bacteria. Mol Microbiol 44:195–204. doi:10.1046/j.1365-2958.2002.02866.x. 11967079

[13] Moric I, Savic M, Ilic-Tomic T, Vojnovic S, Bajkic S, Vasiljevic B 2010 rRNA methyltransferases and their role in resistance to antibiotics. J Med Biochem 29:165–174. doi:10.2478/v10011-010-0030-y.

[14] Mousa JJ, Bruner SD 2016 Structural and mechanistic diversity of multidrug transporters. Nat Prod Rep 33:1255–1267. doi:10.1039/c6np00006a. 27472662

[15] Giedraitienė A, Vitkauskienė A, Naginienė R, Pavilonis A 2011 Antibiotic resistance mechanisms of clinically important bacteria. Medicina (Kaunas) 47:137–146. 21822035

[16] Biggins JB, Onwueme KC, Thorson JS 2003 Resistance to enediyne antitumor antibiotics by CalC self-sacrifice. Science 301:1537–1541. doi:10.1126/science.1086695. 12970566

[17] Galm U, Hager MH, Van Lanen SG, Ju J, Thorson JS, Shen B 2005 Antitumor antibiotics: bleomycin, enediynes, and mitomycin. Chem Rev 105:739–758. doi:10.1021/cr030117g. 15700963

[18] Reimer D, Pos KM, Thines M, Grun P, Bode HB 2011 A natural prodrug activation mechanism in nonribosomal peptide synthesis. Nat Chem Biol 7:888–890. doi:10.1038/nchembio.688. 21926994

[19] Brotherton CA, Balskus EP 2013 A prodrug resistance mechanism is involved in colibactin biosynthesis and cytotoxicity. J Am Chem Soc 135:3359–3362. doi:10.1021/ja312154m. 23406518

[20] Zhang Y, Wen W-H, Pu J-Y, Tang M-C, Zhang L, Peng C, Xu Y, Tang G-L 2018 Extracellularly oxidative activation and inactivation of matured prodrug for cryptic self-resistance in naphthyridinomycin biosynthesis. Proc Natl Acad Sci U S A 115:11232–11237. doi:10.1073/pnas.1800502115. 30327344 PMC6217432

[21] Jiang X, Hashim Ellabaan MM, Charusanti P, Munck C, Blin K, Tong Y, Weber T, Sommer MOA, Lee SY 2017 Dissemination of antibiotic resistance genes from antibiotic producers to pathogens. Nat Commun 8:15784. doi:10.1038/ncomms15784. 28589945 PMC5467266

[22] Napolioni V, Cimarelli L, La Miano A, Teana A, Capuni R, Giuliodori AM, Fabbretti A, Spurio R 2019 Draft genome sequence of Streptomyces sp. AM-2504, identified by 16S rRNA comparative analysis as a Streptomyces kasugaensis strain. Microbiol Resour Announc 8:e00966-19. doi:10.1128/MRA.00966-19. 31537672 PMC6753276

[23] D’Costa VM, McGrann KM, Hughes DW, Wright GD 2006 Sampling the antibiotic resistome. Science 311:374–377. doi:10.1126/science.1120800. 16424339

[24] Perry JA, Westman EL, Wright GD 2014 The antibiotic resistome: what’s new? Curr Opin Microbiol 21:45–50. doi:10.1016/j.mib.2014.09.002. 25280222

[25] Peterson E, Kaur P 2018 Antibiotic resistance mechanisms in bacteria: relationships between resistance determinants of antibiotic producers, environmental bacteria, and clinical pathogens. Front Microbiol 9:2928. doi:10.3389/fmicb.2018.02928. 30555448 PMC6283892

[26] Mak S, Xu Y, Nodwell JR 2014 The expression of antibiotic resistance genes in antibiotic-producing bacteria. Mol Microbiol 93:391–402. doi:10.1111/mmi.12689. 24964724

[27] Cui Z, Wang X-C, Liu X, Lemke A, Koppermann S, Ducho C, Rohr J, Thorson JS, Van Lanen SG 2018 Self-resistance during muraymycin biosynthesis: a complementary nucleotidyltransferase and phosphotransferase with identical modification sites and distinct temporal order. Antimicrob Agents Chemother 62:e00193-18. doi:10.1128/AAC.00193-18. 29735559 PMC6021665

[28] Panter F, Krug D, Baumann S, Müller R 2018 Self-resistance guided genome mining uncovers new topoisomerase inhibitors from myxobacteria. Chem Sci 9:4898–4908. doi:10.1039/c8sc01325j. 29910943 PMC5982219

[29] Auerbach T, Bashan A, Harms J, Schluenzen F, Zarivach R, Bartels H, Agmon I, Kessler M, Pioletti M, Franceschi F, Yonath A 2002 Antibiotics targeting ribosomes: crystallographic studies. Curr Drug Targets Infect Disord 2:169–186. doi:10.2174/1568005023342506. 12462147

[30] Wilson D 2014 Ribosome-targeting antibiotics and mechanisms of bacterial resistance. Nat Rev Microbiol 12:35–48. doi:10.1038/nrmicro3155. 24336183

[31] Sierra RG, Gati C, Laksmono H, Han Dao E, Gul S, Fuller F, Kern J, Chatterjee R, Ibrahim M, Brewster AS, Young ID, Michels-Clark T, Aquila A, Liang M, Hunter MS, Koglin JE, Boutet S, Junco EA, Hayes B, Bogan MJ, Hampton CY, Puglisi EV, Sauter NK, Stan CA, Zouni A, Yano J, Yachandra VK, Soltis SM, Puglisi JD, DeMirci H 2016 Concentric-flow electrokinetic injector enables serial crystallography of ribosome and photosystem II. Nat Methods 13:59–62. doi:10.1038/nmeth.3667. 26619013 PMC4890631

[32] Razi A, Britton RA, Ortega J 2017 The impact of recent improvements in cryo-electron microscopy technology on the understanding of bacterial ribosome assembly. Nucleic Acids Res 45:1027–1040. doi:10.1093/nar/gkw1231. 28180306 PMC5388408

[33] Giuliodori AM, Spurio R, Milon P, Fabbretti A 2018 Antibiotics targeting the 30S ribosomal subunit: a lesson from nature to find and develop new drugs. Curr Top Med Chem 18:2080–2096. doi:10.2174/1568026618666181025092546. 30360712

[34] O’Sullivan ME, Poitevin F, Sierra RG, Gati C, Han Dao E, Rao Y, Aksit F, Ciftci H, Corsepius N, Greenhouse R, Hayes B, Hunter MS, Liang M, McGurk A, Mbgam P, Obrinsky T, Pardo-Avila F, Seaberg MH, Cheng AG, Ricci AJ, Demirci H 2018 Aminoglycoside ribosome interactions reveal novel conformational states at ambient temperature. Nucleic Acids Res 46:9793–9804. doi:10.1093/nar/gky693. 30113694 PMC6182148

[35] Lin J, Zhou D, Steitz TA, Polikanov YS, Gagnon MG 2018 Ribosome-targeting antibiotics: modes of action, mechanisms of resistance, and implications for drug design. Annu Rev Biochem 87:451–478. doi:10.1146/annurev-biochem-062917-011942. 29570352 PMC9176271

[36] Belousoff MJ, Venugopal H, Wright A, Seoner S, Stuart I, Stubenrauch C, Bamert RS, Lupton DW, Lithgow T 2019 cryoEM-guided development of antibiotics for drug-resistant bacteria. ChemMedChem 14:527–531. doi:10.1002/cmdc.201900042. 30667174

[37] Petrelli D, Garofalo C, Lammi M, Spurio R, Pon CL, Gualerzi CO, La Teana A 2003 Mapping the active sites of bacterial translation initiation factor IF3. J Mol Biol 331:541–556. doi:10.1016/s0022-2836(03)00731-9. 12899827

[38] Armstrong CT, Mason PE, Anderson JL, Dempsey CE 2016 Arginine side chain interactions and the role of arginine as a gating charge carrier in voltage sensitive ion channels. Sci Rep 6:21759. doi:10.1038/srep21759. 26899474 PMC4761985

[39] Funatsu G, Wittmann HG 1972 Ribosomal proteins. XXXIII. Location of amino-acid replacements in protein S12 isolated from Escherichia coli mutants resistant to streptomycin. J Mol Biol 68:547–550. doi:10.1016/0022-2836(72)90108-8. 4560854

[40] Shima J, Hesketh A, Okamoto S, Kawamoto S, Ochi K 1996 Induction of actinorhodin production by *rpsL* (encoding ribosomal protein S12) mutations that confer streptomycin resistance in *Streptomyces lividans* and *Streptomyces coelicolor* (A3). J Bacteriol 178:7276–7284. doi:10.1128/jb.178.24.7276-7284.1996. 8955413 PMC178644

[41] Hosaka T, Ohnishi-Kameyama M, Muramatsu H, Murakami K, Tsurumi Y, Kodani S, Yoshida M, Fujie A, Ochi K 2009 Antibacterial discovery in actinomycetes strains with mutations in RNA polymerase or ribosomal protein S12. Nat Biotechnol 27:462–464. doi:10.1038/nbt.1538. 19396160

[42] Aarestrup FM, Jensen LB 2000 Presence of variations in ribosomal protein L16 corresponding to susceptibility of enterococci to oligosaccharides (avilamycin and evernimicin). Antimicrob Agents Chemother 44:3425–3427. doi:10.1128/aac.44.12.3425-3427.2000. 11083650 PMC90215

[43] Adrian PV, Zhao W, Black TA, Shaw KJ, Hare RS, Klugman KP 2000 Mutations in ribosomal protein L16 conferring reduced susceptibility to evernimicin (SCH27899): implications for mechanism of action. Antimicrob Agents Chemother 44:732–738. doi:10.1128/aac.44.3.732-738.2000. 10681347 PMC89755

[44] Kehrenberg C, Schwarz S 2007 Mutations in 16S rRNA and ribosomal protein S5 associated with high-level spectinomycin resistance in *Pasteurella multocida*. Antimicrob Agents Chemother 51:2244–2246. doi:10.1128/AAC.00229-07. 17371823 PMC1891365

[45] Milon P, Konevega AL, Peske F, Fabbretti A, Gualerzi CO, Rodnina MV 2007 Transient kinetics, fluorescence, and FRET in studies of initiation of translation in bacteria. Methods Enzymol 430:1–30. doi:10.1016/S0076-6879(07)30001-3. 17913632

[46] Brandi L, Fabbretti A, Milon P, Carotti M, Pon CL, Gualerzi CO 2007 Methods for identifying compounds that specifically target translation. Methods Enzymol 431:229–267. doi:10.1016/S0076-6879(07)31012-4. 17923238

