# Supplementary figures and images for: Characterization of the Self-Resistance Mechanism to Dityromycin in the Streptomyces Producer Strain
Source: mSphere. 2019 Sep 25;4(5):e00554-19. doi: 10.1128/mSphere.00554-19 (PMC6763770; doi:10.1128/mSphere.00554-19)

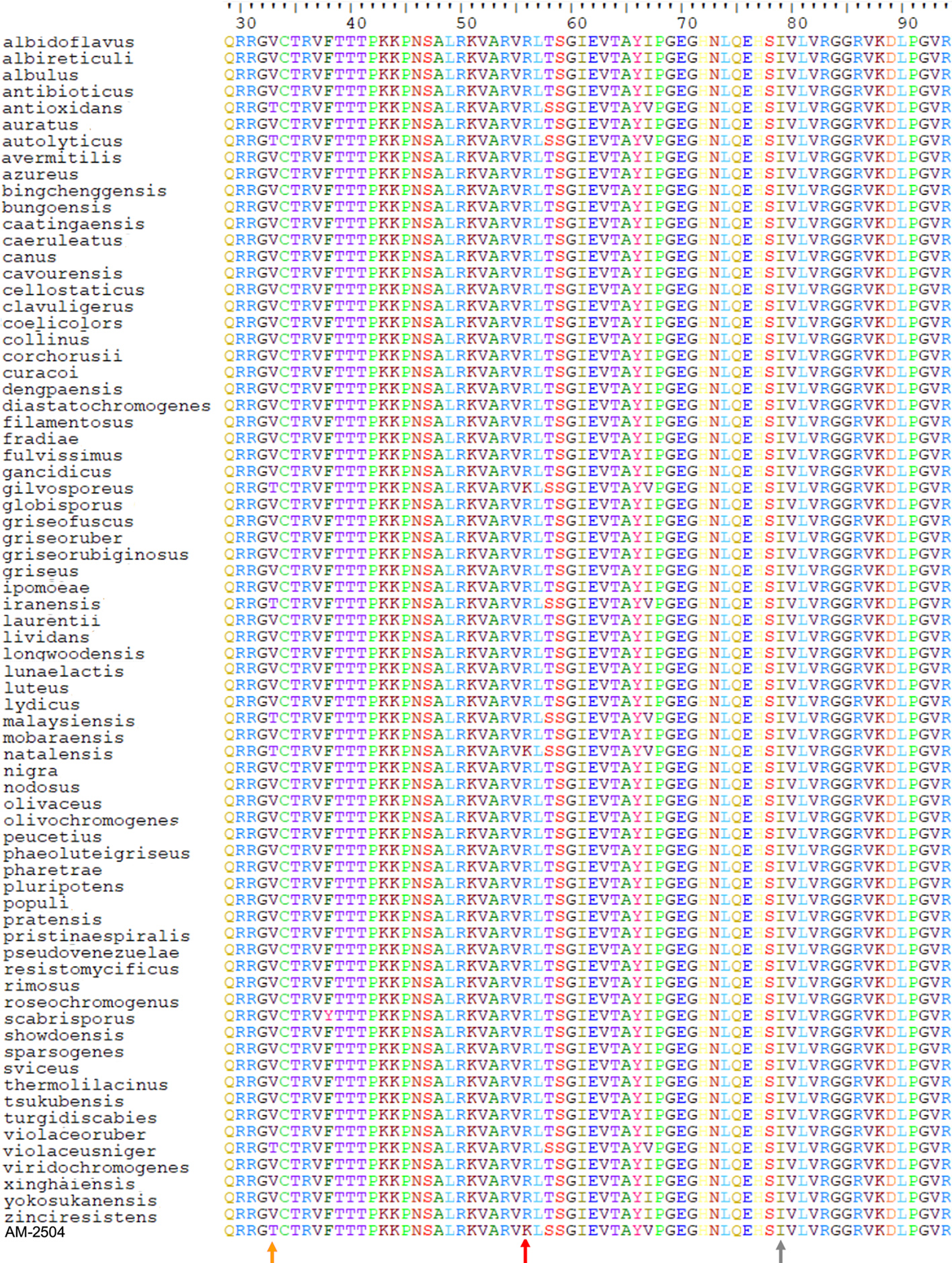

Supplement: FIG S1 [file mSphere.00554-19-sf001.jpg]

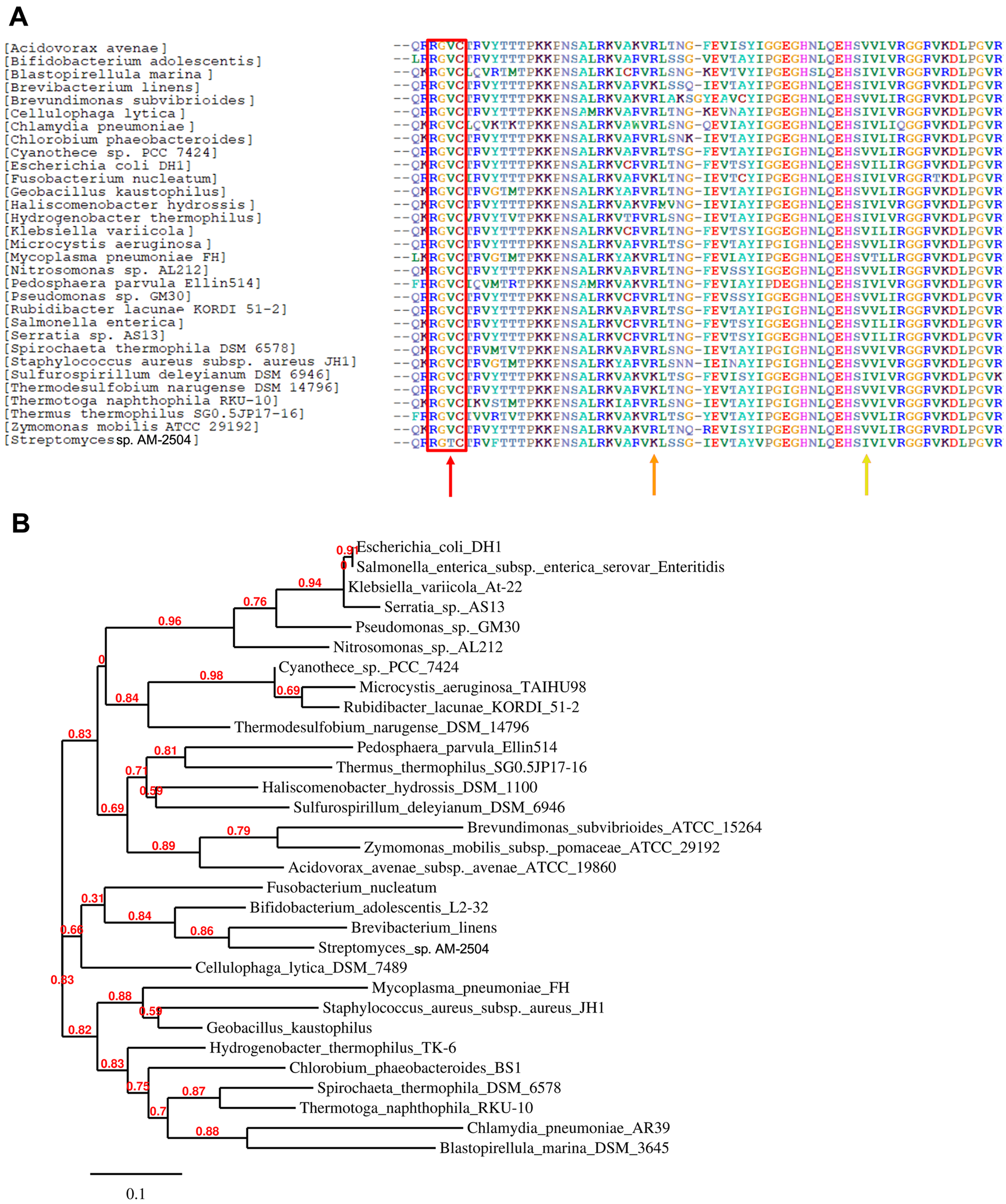

Supplement: FIG S2 [file mSphere.00554-19-sf002.tif]
